# Cofilin/Twinstar Phosphorylation Levels Increase in Response to Impaired Coenzyme A Metabolism

**DOI:** 10.1371/journal.pone.0043145

**Published:** 2012-08-17

**Authors:** Katarzyna Siudeja, Nicola A. Grzeschik, Anil Rana, Jannie de Jong, Ody C. M. Sibon

**Affiliations:** Department of Cell Biology, Radiation and Stress Cell Biology, University Medical Center Groningen, University of Groningen, Groningen, The Netherlands; Instituto Gulbenkian de Ciência, Portugal

## Abstract

Coenzyme A (CoA) is a pantothenic acid-derived metabolite essential for many fundamental cellular processes including energy, lipid and amino acid metabolism. Pantothenate kinase (PANK), which catalyses the first step in the conversion of pantothenic acid to CoA, has been associated with a rare neurodegenerative disorder PKAN. However, the consequences of impaired PANK activity are poorly understood. Here we use *Drosophila* and human neuronal cell cultures to show how PANK deficiency leads to abnormalities in F-actin organization. Cells with reduced PANK activity are characterized by abnormally high levels of phosphorylated cofilin, a conserved actin filament severing protein. The increased levels of phospho-cofilin coincide with morphological changes of PANK-deficient *Drosophila* S2 cells and human neuronal SHSY-5Y cells. The latter exhibit also markedly reduced ability to form neurites in culture – a process that is strongly dependent on actin remodeling. Our results reveal a novel and conserved link between a metabolic biosynthesis pathway, and regulation of cellular actin dynamics.

## Introduction

Regulation of actin dynamics is critical for cellular function. Cells respond to various external and internal stimuli by specific remodeling events of the actin cytoskeleton. Actin rearrangements are required for changes in cell shapes and thus actin dynamics are important for a variety of morphogenetic events such as cell divisions, cell migration, adhesion, neuritogenesis (sprouting of neurites) or axon pathfinding. At the same time, the regulation of these processes is tightly linked to the metabolic status of cells and organisms. Numerous data demonstrate the involvement of Rho family GTPases in the control of actin filament nucleation and disassembly [1], [2], however relatively little is known about whether and how actin cytoskeleton signaling is influenced by and tuned with the metabolic state of the cell.

Coenzyme A (CoA) is an central metabolite present in all living organisms [3]. CoA reacts with carboxyl groups giving rise to thioesters – CoA-activated acyl moieties. About 4% of all known enzymes utilize CoA as a cofactor and CoA thioesters are essential for over 100 different reactions of the intermediary metabolism, such as the tricarboxylic acid cycle (TCA cycle), lipid synthesis and oxidation or the synthesis of some amino acids (reviewed in [3]). Hence, CoA occupies a central position in the regulation of the cellular metabolism. One evolutionary conserved pathway has been described leading to the *de novo* biosynthesis of CoA. The first step of this pathway is phosphorylation of pantothenic acid (vitamin B5) by pantothenate kinase (PANK). The pathway has gained renewed attention after the discovery that mutations in one of the four human PANK isoforms, PANK2, lead to a severe neurodegenerative disorder, Pantothenate Kinase-Associated Neurodegeneration (PKAN) [4]. Recently, we and others have established a *Drosophila melanogaster* model for PKAN [5], [6], [7], [8] and we have shown that *Drosophila dPANK/fbl* mutant flies as well as downregulating dPANK/Fbl using an *in vitro* RNAi approach in *Drosophila* Schneider's S2 cells constitute suitable models for studying the consequences of CoA deficiency. The *dPANK/fbl* gene was initially identified in a screen for male sterility and *dPANK/fbl* mutants show cell division errors and cytokinesis defects with abnormal F-actin dynamics [5], [8]. Additionally, abnormal F-actin accumulation is observed in the ovaries of CoA deficient female flies, which are also sterile [9]. This correlation of CoA metabolism in flies with actin related processes suggests additional, yet not appreciated, influences of CoA levels. Nevertheless the molecular mechanisms of these CoA-related actin abnormalities are not known.

Here we use *Drosophila* S2 cells to study in more detail actin defects caused by CoA deficiency. We demonstrate that phosphorylation of a *Drosophila* homolog of cofilin, Twinstar (Tsr) [10], is increased in CoA deficient cells. Cofilin is an actin binding protein influencing depolymerization and severing of actin filaments and it plays an essential role in F-actin turnover [11], [12] and is has been reported that activity of cofilin is inhibited by phosphorylation [13], [14], [15]. Our study reveals involvement of Cdi kinase and Slingshot phosphatase in the regulation of actin dynamics in *Drosophila*, in response to changes in CoA metabolism. Furthermore we show that regulation of cofilin in response to impaired CoA biosynthesis is conserved between *Drosophila* cells and human neuronal cells. By inhibiting PANK activity during neuronal differentiation *in vitro,* not only the phosporylation status of human cofilin was affected but also the cellular morphology and neurite formation – processes which are strongly dependent on actin remodeling.

## Results

### dPANK/Fbl downregulation leads to actin abnormalities in *Drosophila* S2 cells

**Figure 1:**
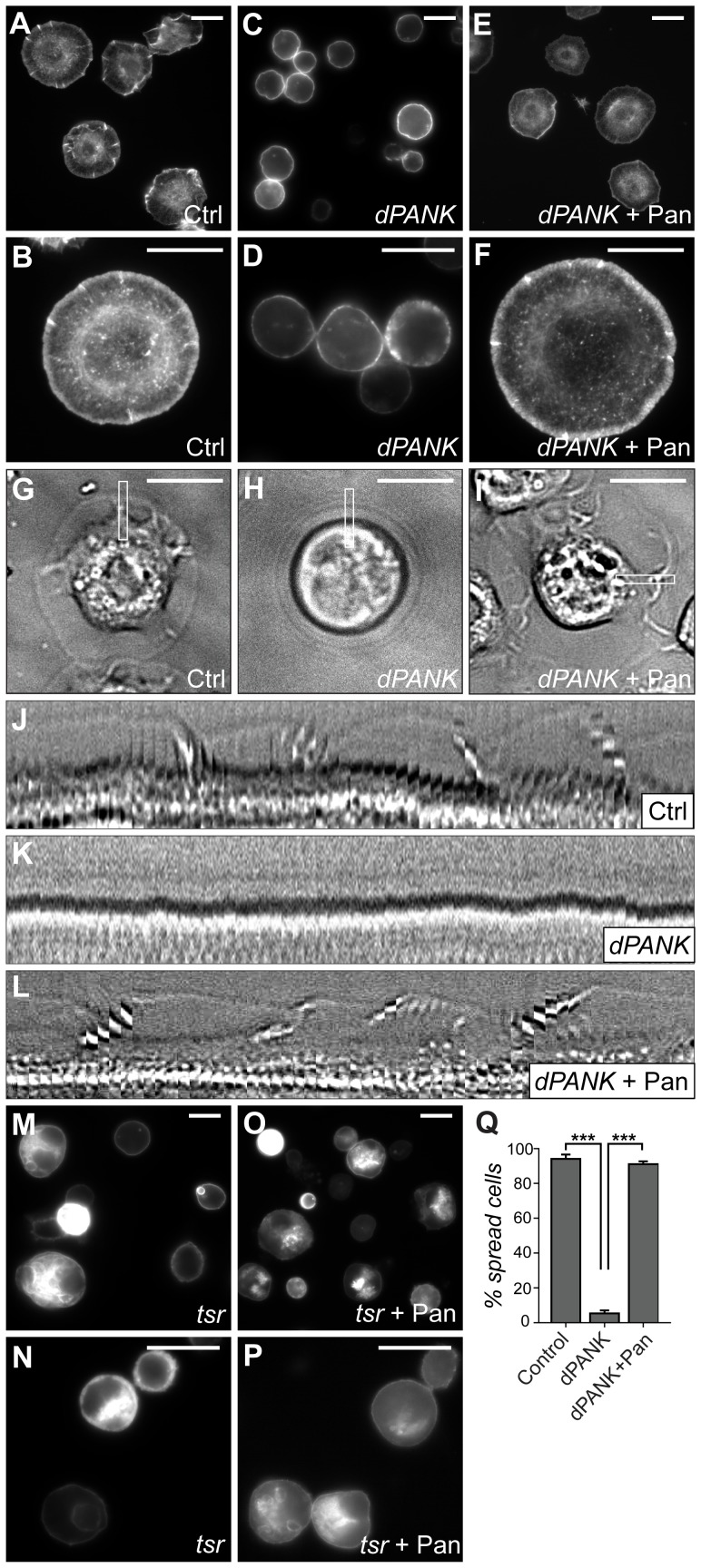
CoA deficient *Drosophila* S2 cells fail to form actin-based lamella. S2 cells were seeded on concanavalin A to induce cell spreading and lamella formation. Actin was visualized with rhodamine-phalloidin staining. (**A,B**) Representative images of the Ctrl; (**C,D**) dPANK/Fbl RNAi; (**E,F**) dPANK/Fbl RNAi treated cells supplemented with pantethine; (**M,N**) Twinstar RNAi; and (**O,P**) Twinstar (Tsr) RNAi treated cells supplemented with pantethine are shown. (**G–L**) S2 cells were seeded in concanavalin A coated glass bottom culture dishes and imaged during cell spreading and lamella formation in phase contrast with an inverted Zeiss confocal microscope using a 63× glycerol-immersion lens. Frames were acquired every 20 seconds for 20 minutes. Individual images from Ctrl (**G**), dPANK/Fbl RNAi (**H**), dPANK/Fbl RNAi treated cells supplemented with pantethine (**I**) are shown. Images are representative of at least 5 movies taken in different regions of each dish. The white box in all three images depicts the area used to create the kymographs in **J–L**. (**J**) Kymographs of Ctrl cells; (**K**) dPANK/Fbl RNAi; (**L**) dPANK/Fbl RNAi treated cells supplemented with pantethine. The kymographs were generated from an area transecting the cell membrane (outlined by a white box in **G–I**) and correspond to the movies from which the images were taken (for movies see supplementary **Figure S4, S5, S6, S7**). (**Q**) Quantification of the percentage of spread (lamella forming) cells as illustrated in A–L. Scale bars represent 10 µm. Ctrl = control/non-treated cells; Pan = pantethine treatment; dPANK = dPANK/fbl RNAi treated cells; tsr = twinstar RNAi treated cells.

In *Drosophila* pantothenate kinase is encoded by a single gene *dPANK/fbl* [8]. Decreased expression of dPANK/Fbl protein leads to a dramatic reduction in the levels of total CoA both in *Drosophila dPANK/fbl* mutant flies and Schneider's S2 cells [6]. Interestingly, *dPANK/fbl* mutant flies show actin abnormalities during spermatogenesis and oogenesis [8], [9]. To elucidate the link between CoA metabolism and actin cytoskeleton, we first investigated whether RNAi mediated downregulation of dPANK/Fbl in S2 cells also leads to F-actin abnormalities. *Drosophila* Schneider's cells have been successfully used to identify new players involved in actin cytoskeleton dynamics and regulation of cell morphology [16], [17]. Hence, we plated control and dPANK/Fbl downregulated S2 cells on concanavalin A-coated glass coverslips to induce cell flattening and spreading [16]. As revealed by phalloidin staining, control cells showed the characteristic morphology with lamella formation, whereas CoA deprived cells failed to undergo morphological changes under these conditions (**Figure 1A, B, C, D** **,** for quantification see **Figure 1Q**). Impaired function of various proteins involved in formation of actin-based lamella is associated with specific morphological changes in S2 cells [16]. We have compared the morphology of CoA deficient cells with these reported abnormalities and noticed similarities between dPANK/Fbl RNAi-treated cells and cells depleted of the *Drosophila* ADF/cofilin homolog, Twinstar (Tsr) (**Figure 1M,N**). Next we assayed the dynamics of the lamella by making time-lapse recordings using phase contrast microscopy (for movies see **Movie S1, S2, S3, S4**) (Rogers *et al.,* 2003). Control cells plated on concanavalin A show dynamic behavior of lamella (**Figure 1G,J**) whereas dPANK/Fbl RNAi-treated cells do not form the lamella (**Figure 1H,K**). Further, we asked whether observed actin abnormalities where indeed the consequence of decreased cellular CoA levels or were due to the absence of dPANK/Fbl protein. Previously we showed that addition of pantethine to the cell culture medium restored CoA levels in the presence of strongly reduced levels of dPANK/Fbl protein [6](Siudeja et al., 2011). Pantethine supplementation reversed the morphological abnormalities of dPANK/Fbl depleted cells (**Figure 1E,F,I,L** for quantification see **Figure 1Q**), confirming that decreased CoA levels are strongly associated with a failure of S2 cells to spread on concanavalin A coated surface and are strongly associated with abnormal actin based lamella. Addition of pantethine to Tsr RNAi-treated cells did not rescue the phenotype (**Figure 1O,P**), further proving that the beneficial effect of pantethine in dPANK/Fbl RNAi cells is due to the restoration of CoA levels in these cells, and not due some general (CoA-unrelated) positive effects of pantethine on the actin cytoskeleton.

### Twinstar phosphorylation is increased in CoA deficient cells

**Figure 2:**
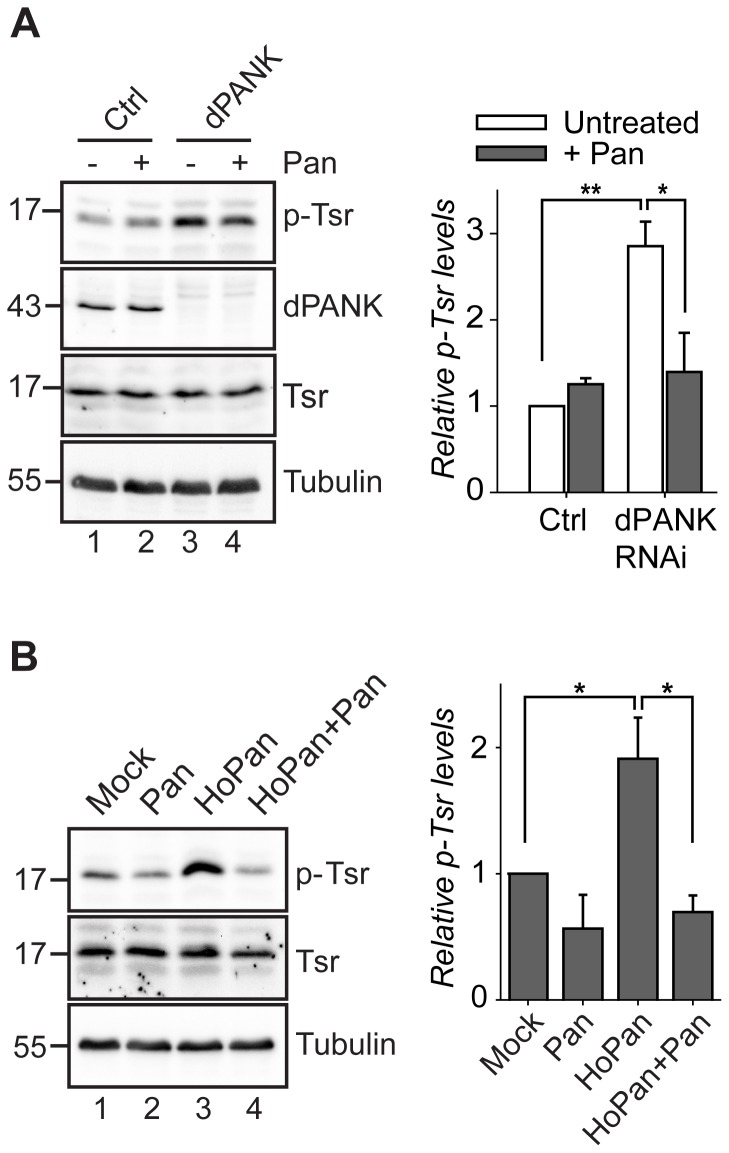
Inhibition of dPANK activity in S2 cells induces an increase in phospho-Twinstar levels. Immunoblots of whole cell extracts were incubated with an antibody against Tsr and p-Tsr. Tubulin was used as a loading control. (**A**) Cells were treated with control dsRNA or dsRNA to downregulate dPANK/Fbl protein levels and medium was supplemented with pantethine when indicated. The graph illustrates the quantified levels of p-Tsr relative to untreated control cells. (**B**) S2 cells were cultured in the presence of pantethine, HoPan or both compounds. The graph illustrates the quantified levels of p-Tsr relative to untreated control cells.

The similarity in morphology between dPANK/Fbl depleted and Tsr depleted cells prompted us to hypothesize that Tsr could be involved in the actin abnormalities in the CoA deficient background. Tsr is essential for actin remodeling in *Drosophila*, both *in vivo* and *in vitro* [10], [16], [18], [19]. The actin binding and severing activity of Tsr is inhibited by phosphorylation at the conserved serine residue 3. Thus, using specific antibodies [20], we investigated levels of phopsho-Twinstar (p-Tsr) in CoA-deficient S2 cells. Western blot analysis revealed a significant increase in dPANK/Fbl RNAi-treated cells as compared to control cells (**Figure 2A**). Further, we used a non-genetic approach to block the CoA biosynthesis pathway by using the pantothenate kinase inhibitor HoPan [21]. HoPan treatment resulted also in an increase in p-Tsr levels in S2 cells (**Figure 2B**). Increased phosphorylation of Tsr is already observed after 4 days dPANK/fbl RNAi treatment (**Figure S1**). The increase in Tsr phosphorylation after 4 days dPANK/Fbl RNAi was rescued by the addition of pantethine (during the following 3 days), demonstrating that in S2 cells Tsr phosphorylation levels are responsive to changes in CoA levels and do not respond to the levels of the PANK protein itself (Figure 2). These results also indicate that increased phosphorylation of Tsr in response to decreased CoA levels is reversible upon restoration of CoA levels. Increased phosphorylation of Tsr upon HoPan treatment is also rescued by pantethine (**Figure 2B**).

These data, together with our morphological studies (**Figure 1**) strongly suggest that decreased CoA levels are associated with hyperphosphorylation (and inhibition) of Tsr, leading to the observed actin abnormalities.

Next we investigated whether the increased levels of p-Tsr are a general response to metabolic stress. Hereto, we serum starved the cells and we blocked ATP synthesis with oligomycin. Increased phosphorylation of AMP-activated kinase confirmed these stress conditions [22], [23] (**Figure S2**). Under these metabolic stress conditions, levels of p-Tsr did not change (upon serum starvation) or showed a decrease (upon incubation with oligomycin) (**Figure S2**). In addition serum starvation did not induce changes in actin lamellipodia homeostasis in *Drosophila* S2 cells [20]. These results demonstrate that by decreasing CoA levels a specific cellular response is provoked, different from a general reaction induced by starvation.

### Cdi kinase and Slingshot phosphatase are involved in Twinstar hyperphosphorylation associated with decreased CoA levels

**Figure 3:**
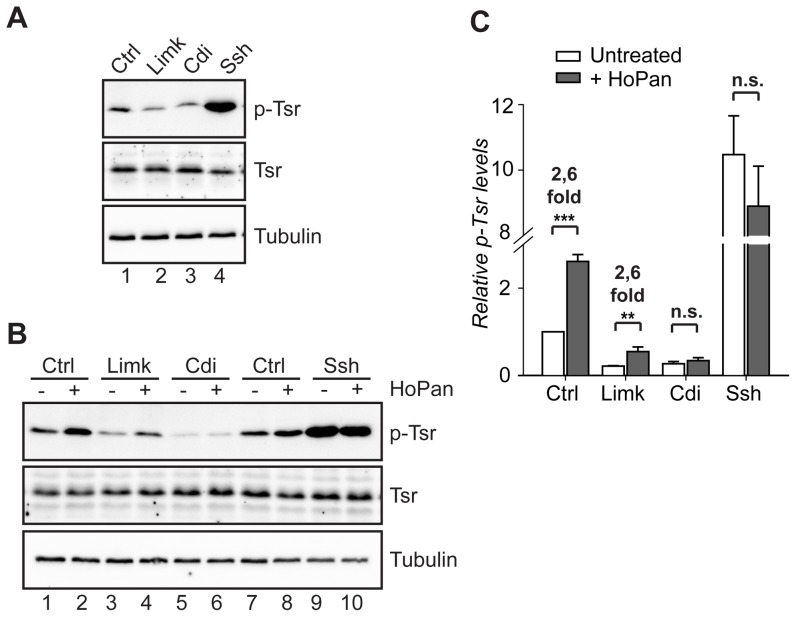
Cdi and Ssh signaling is involved in the regulation of Twinstar in response to decreased levels of CoA. (**A**) *Drosophila* LIM kinases, dLimk, Center divider (Cdi), and Slingshot phosphates (Ssh) regulate levels of p-Tsr in S2 cells. Cells were treated with dsRNA against dLimk, Cdi, Ssh or with control dsRNA and the levels of Tsr and p-Tsr were assayed using Western blot analysis. Tubulin was used as a loading control. (**B**) dLimk, Cdi or Ssh were down-regulated for 3 days after which cells were subcultured and left untreated or HoPan was added to the cell culture medium. After additional 48 hr cell lysates were assayed for levels of Tsr and levels of p-Tsr. (**C**) Quantification of the levels of p-Tsr as illustrated in B. Fold change after HoPan addition is indicated above the bars, n.s: not significant.

Having established that CoA deficiency correlates with defects in F-actin organization and with an increase in accumulation of the phosphorylated form of Tsr, we further aimed to identify upstream mediators of this response. Cofilin phosphorylation is catalyzed by a family of LIM kinases [13], [15]. In *Drosophila* two kinases belonging to this group have been characterized, dLimk - the ortholog of human LIM kinase [24] and Center divider (Cdi) - orthologus to human testicular kinase 1 (TESK1) [25]. Further, the reactivation of cofilin is achieved by dephosphorylation catalyzed by Slingshot (Ssh) phosphatases [14]. Hence, by using an RNAi-approach we tested whether the accumulation of p-Tsr in a CoA deficient background is mediated by the above-mentioned players. Consistent with previous reports [20], RNAi induced silencing of either dLimk or Cdi resulted in a decrease in basal Tsr phosphorylation levels, whereas a knockdown of Ssh phosphatase induced the opposite effect (**Figure 3A**). Interestingly, cells expressing reduced levels of dLimk still showed an increase in p-Tsr levels in response to decreased CoA levels caused by HoPan treatment. Conversely, cells expressing reduced levels of Cdi kinase or Ssh did not show any change in the levels of p-Tsr upon incubation with HoPan (**Figure 3B and C**). These results implicate that normal levels of both Cdi kinase and Slingshot phosphatase are required for increased p-Tsr levels in response to impaired CoA metabolism. The two-fold induction in Tsr phosphorylation in the presence of reduced levels of dLimk indicate that either the observed phosphorylation is dLimk independent or reduced levels of dLimk are still sufficient to induce Tsr phosphorylation.

All together these results suggest that in *Drosophila* S2 cells Tsr regulation in response to fluctuating CoA levels is mediated by pathways, which include Cdi kinase and Slingshot phosphatase activity, both of which are known regulators of the cofilin phospho-status.

### PANK inhibition inactivates mammalian cofilin and affects neuritogenesis *in vitro*

**Figure 4:**
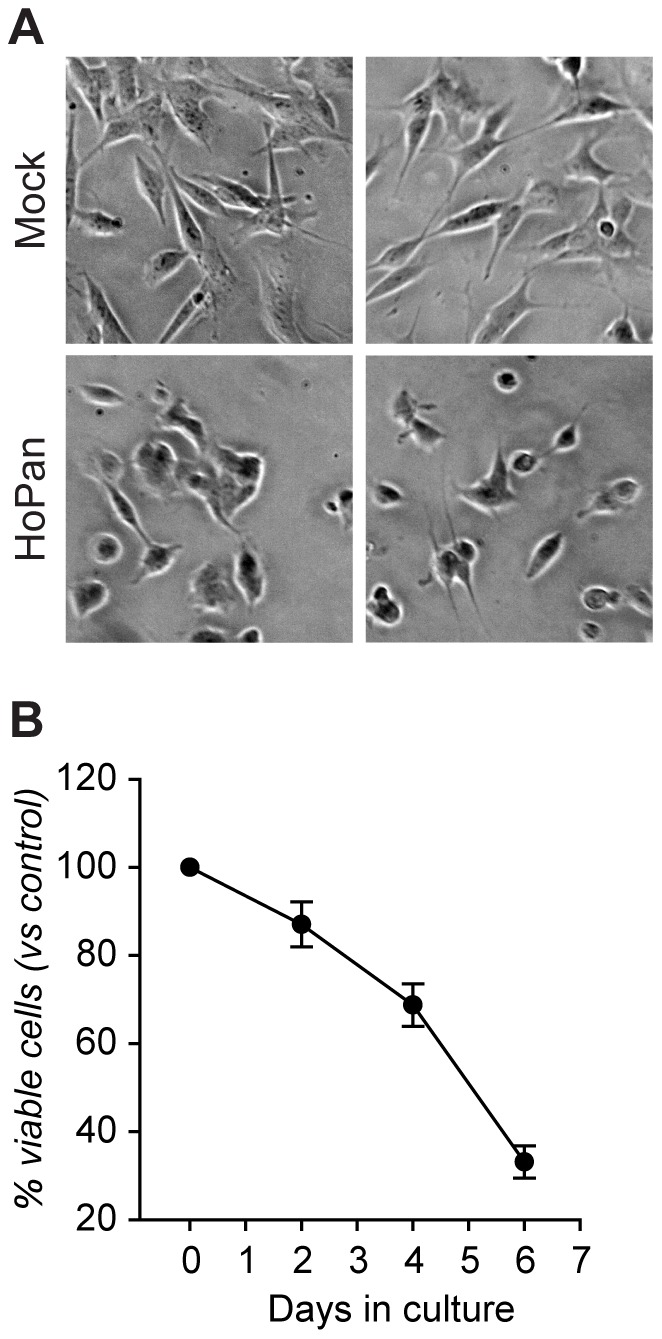
The morphology and viability of cultured human neuroblastoma SHSY-5Y cells is affected upon PANK inhibition. SHSY-5Y cells were cultured in medium supplemented with 0,5 mM HoPan. (**A**) After 6 days in culture the cell morphology was visualized with an inverted microscope. Representative images of control (mock treated) and HoPan treated cells are shown. (**B**) Cell viability after 0–6 days in culture with HoPan was assayed by a Trypan blue exclusion test. Viability of control untreated cells was set as 100% at each time point.

**Figure 5:**
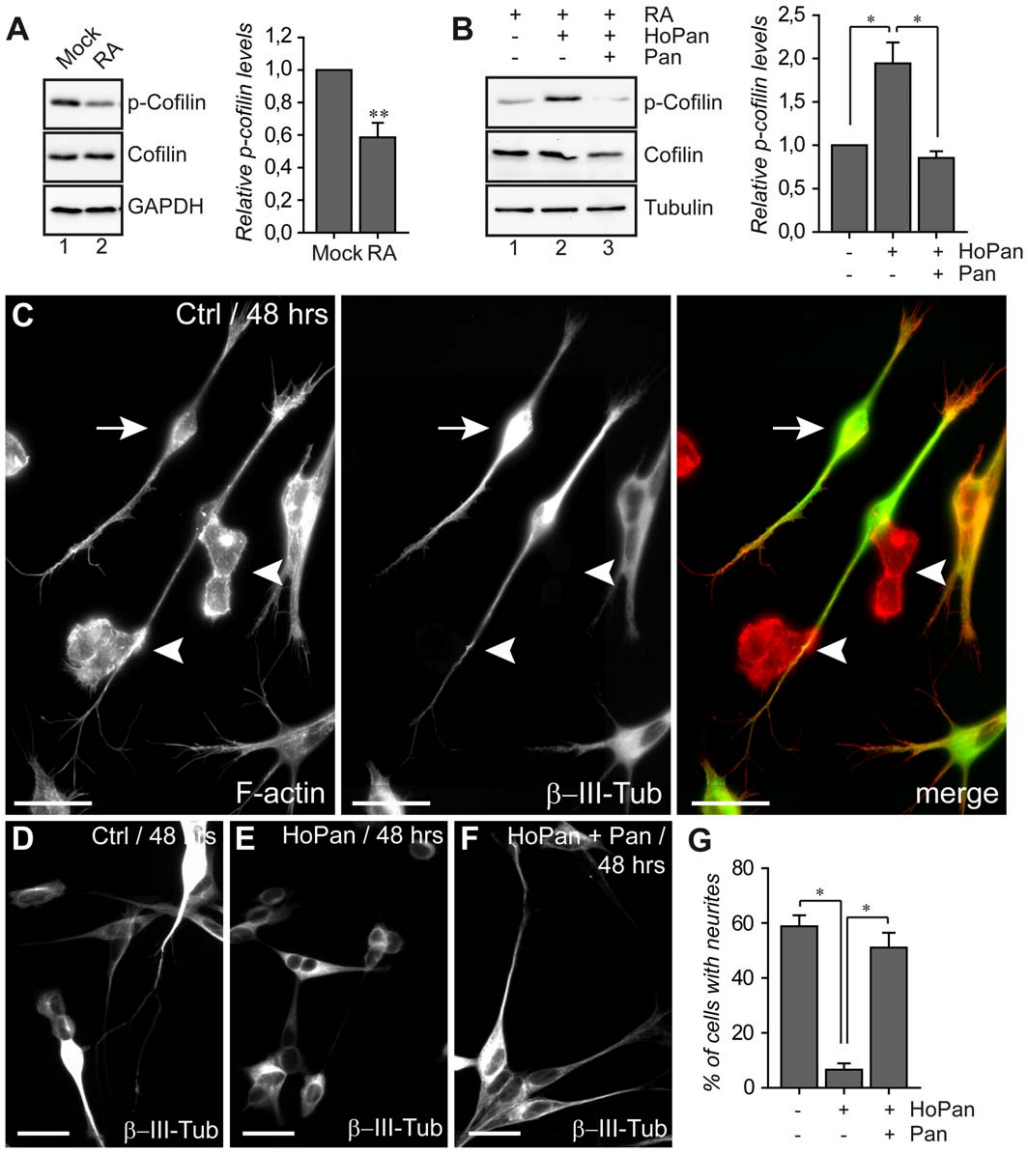
HoPan induces accumulation of phospho-cofilin and affects neurite formation in neuronal differentiation *in vitro*. (**A**) Undifferentiated and retinoic-acid (RA) differentiated SHSY-5Y cells were lysed and levels of total and phosphorylated-cofilin were assayed using Western blot analysis with specific antibodies. Representative blots are shown. GAPDH was used as an additional loading control. Levels of p-cofilin were quantified from 2 independent experiments. The p-cofilin/cofilin ratio was set as 1 in mock treated cells. (**B**) RA-differentiated SHSY-5Y cells were additionally treated with HoPan or HoPan and pantethine. Levels of p-cofilin were assayed and quantified as in A. (**C**) Control SHSY-5Y cells were differentiated with RA, plated on poly-lysine and analyzed after 48 hours for their morphology (using rhodamin-phalloidin marking F-actin, red) and differentiation status (using β-III-Tubulin staining, green). In RA-treated cultures undifferentiated and differentiated cells are visible. Undifferentiated cells showed very short filopodia and did not express β-III-Tubulin (indicated as arrowheads). 48 hours after seeding many differentiated cells showed an elongated shape, neurite growth and high expression of β-III-Tubulin (indicated as arrows). (**D–F**) Control (D), HoPan (E) or HoPan plus pantethine (F) treated cells were differentiated with RA and plated on poly-lysine. 48 hours after seeding cells were fixed and stained for β-III-tubulin, representative parts of cell morphology and β-III-Tubulin staining of the different conditions are shown. The percentage of neurite-bearing cells was calculated for each condition. Scale bars represent 25 µm (**C–F**). (**G**) Quantification of cells showing β-III-Tubulin positive neurites is presented.

In the experiments as shown above we demonstrate that interfering with the pantothenate kinase activity in *Drosophila* S2 cells results in the failure to form actin-based lamella and in the accumulation of phosphorylated (and most likely inactive) Tsr – the *Drosophila* ortholog of cofilin. In humans mutations in the *PANK2* gene cause PKAN – a severe neurodegenerative disorder with a largely unresolved pathophysiology. The regulation of cytoskeletal dynamics plays a fundamental role in neuronal function and it is especially important in the process of neuritogenesis [26], [27]. Furthermore, cofilin (together with Lim kinases and Slingshot) appears to be a key regulator of actin cytoskeleton dynamics during growth cone formation and neurite extension [28], [29], [30], [31], [32]. Therefore, we investigated whether inactivation of PANK activity in human neuronal cells leads to changes in the phosphorylation status of cofilin and whether this correlates with altered neuronal morphology. First, we cultured undifferentiated neuroblastoma SHSY-5Y cells during four doubling times with addition of HoPan in the cell culture medium in order to block the enzymatic activity of PANK. Under these conditions of HoPan treatment reduced cell counts and abnormal morphology was observed (**Figure 4A and B**). In addition, HoPan also induced a detachment of the treated cells from the culture dishes. This clearly indicates that, similarly to *Drosophila* S2 cells, impaired CoA biosynthesis affects survival and morphology of dividing human neuroblastoma cells. Further we used retinoic acid (RA) to differentiate SHSY-5Y cells [33], [34] and to investigate the effects of HoPan on neurite formation. First we examined cofilin phosphorylation levels upon differentiation of SHSY-5Y cells. Western blot analysis revealed that treatment with RA resulted in a significant decrease in phospho-cofilin levels as compared to mock treated cells (**Figure 5A**). Further, we compared the levels of phospho-cofilin in differentiated SHSY-5Y cells non-treated or treated with HoPan. Similarly to our results obtained with *Drosophila* cells, HoPan induced an accumulation of the phosphorylated cofilin in human SHSY-5Y cells (**Figure 5B**). Finally, we investigated the effect of HoPan treatment on the morphology and differentiation of RA treated SHSY-5Y cells (see also **Figure S3**). Firstly, control SHSY-5Y cells were differentiated with RA, plated on poly-lysine and analyzed after 24 hours and 48 hours. Morphology was investigated by F-actin staining and differentiation status was analyzed by using β -III-Tubulin – a neuronal specific isoform of tubulin [35], [36], [37] as a marker for differentiation. In RA treated cultures (at 24 and 48 hours) three classes of cells were observed. The first class of cells displayed a round shape; they were β-III-Tubulin negative, with low F-actin and only short (if any) filopodia. These cells were classified as not-differentiated (arrowheads **Figure 5C**; **Figure S3A**). The second class of cells had a spread, angular appearance. Strong, filamentous actin fibers were present and a low expression of β-III-Tubulin could be detected (**Figure S3B, C**). The third class of cells, mainly detected 48 hours after seeding had an elongated shape with rather small cell body, accompanied by high levels of F-actin. They showed strong neurite growth and high expression of β-III-Tubulin (**Figure 5C** **,** arrows). They were scored as fully differentiated neurite-bearing cells. No β-III-Tubulin expressing cells (class 2 and 3) were observed in the SHSY-5Y cell culture not treated with RA.

48 hours after RA treatment, 60% of the control cells were fully differentiated and showed neurite growth and a high expression of β-III-Tubulin (class 3, **Figure 5C, D**). In contrast HoPan treated cells showed a low (less than 10%) percentage of cells with high β-III-Tubulin expression and neurite formation (**Figure 5E**). Finally, we tested whether the HoPan-induced phenotypes in SHSY-5Y cells could be reverted by the addition of pantethine to the cell culture medium. The rescue by pantethine was evident for all the phenotypes investigated (**Figure 5B, F, G**). In RA treated cultures, RA and HoPaN treated cultures and RA, HoPan and pantetehine treated cultures, all three classes of cells were present, however the amount of class 3 cells was strikingly different. **Figure 5D, E, F** shows representative images of the conditions used and quantification is presented in **Figure 5G**. These results strongly suggest that the observed defects are due to decreased levels of CoA and not due to some non-specific (and non-PANK related) effect of HoPan. These results indicate that accumulation of phosphorylated (and most likely inactive) form of cofilin under conditions of impaired CoA biosynthesis coincides with impairment of neurite formation *in vitro* –a process which is strongly dependent on actin remodeling.

## Discussion

The actin cytoskeleton mediates a variety of essential biological functions in all eukaryotic cells. The molecular mechanisms responsible for actin remodeling have been extensively studied. However, relatively little is known how cellular metabolism influences cell morphology and cytoskeleton dynamics. Recent studies convincingly show that these events are closely linked. For example, it is well recognized that insulin and growth factor signaling induces actin filament remodeling promoting membrane ruffling in diverse mammalian cell types [38], [39]. In *Drosophila* S2 cells F-actin rich lamellapodia are also being formed in response to insulin-induced Akt signaling [20]. Further, studies using *Drosophila* and mammalian systems suggest that energy metabolism is directly linked to cell structure regulation via the Lkb1/AMP-activated protein kinase (AMPK) signaling pathway [40], [41], [42], [43]. The physiological importance of this crosstalk between metabolism and the cytoskeleton remains poorly understood but relevant to investigate because it may contribute to the understanding of specific pathological conditions. For example Lkb1, which plays a central role in energy metabolism and actin filaments assembly [44] is linked to tumorigenesis. Mutations in the human *LKB1* gene result in increased incidence of epithelial cancer [45], which suggests that loss of Lkb1 in epithelia may contribute to the tumorigenic process through effects on the actin cytoskeleton. Disturbance of the tight link between the cellular metabolic status and regulation of the actin cytoskeleton may also underlie a number of metabolic diseases with an unresolved pathophysiology.

PKAN is a severe neurodegenerative disease caused by mutations in the human pantothenate kinase 2 (PANK2)-encoding gene and the molecular basis of this devastating disease are largely unknown [4]. Previously we have established *Drosophila* and human cell line models for PKAN, in which PANK activity is impaired [5], [6]. Here, we demonstrate that impaired CoA metabolism influences actin associated cellular events, such as the regulation of the cell shape. We show that inhibition of PANK activity results in changes in cell morphology in *Drosophila* as well as in human neuronal cell cultures. Further we identify a conserved mechanism of cofilin/Twinstar inactivation in response to decreased CoA levels. We show that in *Drosophila* the inability of CoA-deficient S2 cells to form actin rich lamellipodia, correlates with an increase in phospho-Tsr levels and Cdi kinase and slingshot phosphatase are involved in this increased phosphoraylation. These results underscore previously published data that in general cofilin is an important regulator of actin dynamics (reviewed in [46]) and are in agreement with data showing that increased Tsr phosporylation is associated with the inability of S2 cells to form actin-based lammelipodia [20]. It is currently unclear whether decreased levels of CoA influence kinase activity of Cdi or phosphatase activity of Slingshot. Alternatively, Tsr itself could be modified in the presence of decreased CoA levels in such a way that it is being more efficiently phosphorylated or less efficiently dephosphorylated.

Finally, in our current work we show that not only *Drosophila* cells but also differentiated human neuronal cells show abnormal F-actin organization and an increase in phospho-cofilin levels upon impaired PANK function. Differentiated neuroblastoma SHSY-5Y cells undergo extensive morphological changes, which finally lead to the formation of neurites [34]. During these morphological changes, we observed that cofilin is maintained in a non-phosphorylated (active) state. The latter is most likely required for high actin filament dynamics, necessary to undergo the morphogenesis. Analogous involvement of cofilin in neuronal differentiation has also been reported both in primary cultures (such as isolated chicken neurons) and in other cell lines (such as PC12 cells) [28], [29], [30], [31], [32]. We show that the addition of PANK inhibitor, HoPan, to the medium not only inactivates cofilin but also affects the ability of differentiated SHSY-5Y cells to interact with tissue culture surface and the ability to form neurites.

The physiological importance of actin regulation in neuronal function *in vivo* can be illustrated by a contribution of actin signaling pathways to brain disorders, especially to mental retardation [47], [48]. Moreover cofilin activity itself has recently been reported to regulate neuronal migration [49], spine morphology [50] and synaptic plasticity [51] in mice. In humans, abnormal expression of LIM-kinase1 is associated with a mental disorder with profound cognition deficits [52]. Together these and our studies underscore the physiological importance of actin regulation and suggest that mutations that disrupt normal PANK activity (as in PKAN) can lead to altered actin dynamics and neuronal dysfunction. It needs to be pointed out that altered CoA metabolism does also influence other cytoskeletal components because recently we demonstrated that tubulin acetylation is strongly decreased in *Drosophila* and human cell models of PKAN (Siudeja *et al*., 2011). However, cell spreading and neurite formation (as measured here) depend mostly on the reorganization of the actin cytoskeleton, whereas microtubule polymerization is required for the elongation of already existing neurites [26], [53]. Additional studies will be required to investigate whether cofilin inactivation and/or actin abnormalities indeed underlie the pathology of PKAN, although at present these experiments are hindered by a very limited access to patient-derived material.

Surprisingly little is known about the implications of altered *de novo* CoA synthesis in higher eukaryotes and our results reveal a novel and conserved link between CoA metabolism and the regulation of actin dynamics. We address, for the first time, the consequences of impaired PANK function on actin organization and dynamics and the results obtained contribute to the general cell biology knowledge and may potentially increase our understanding of the human neurodegenerative disorder PKAN.

## Materials and Methods

### Drosophila cell culture - Drosophila Schneider's S2 cells were maintained at 25°C in Schneider's Drosophila medium

(Invitrogen) supplemented with 10% heat inactivated fetal calf serum (Gibco) and antibiotics (penicillin/streptomycin, Invitrogen). Cells in the exponential phase of growth were used for all the experiments. When required 0,5 mM HoPan (Zhou Fang Pharm Chemical, Shanghai, China) and/or 0,1 mM pantethine were added directly to the cell culture medium. HoPan treatment was continued for 2–3 days.

### dsRNA synthesis and RNAi

T7-flanked primer sequences were designed to amplify gene specific DNA templates (for primer sequences, see **Table S1**). DNA templates were amplified using standard PCR methods. Double-stranded RNAs were obtained by *in vitro* transcription and purified using MegaScript RNAi Kit (Ambion). 2 pmols of DNA templates were used per 20 µL *in vitro* transcription reaction. dsRNA treatment was carried out as described previously [54]. Cells were incubated in serum-free medium containing 40 nM dsRNA for 1 hour, following the addition of serum containing medium. The cells were incubated for 4 days to induce an efficient knock-down. Cells were subcultured, drugs (HoPan and/or pantethine) were added to the medium and the cells were maintained for additional 2–3 days until analysis.

### SHSY-5Y culturing and differentiation

SHSY-5Y human neuroblastoma cells were maintained in dMEM (Invitrogen) supplemented with 10% FCS (Gibco) and antibiotics (penicillin/streptomycin, Invitrogen). Cells of a passage between 10–30 were used. For HoPan treatment, cells were cultured in custom made dMEM without vitamin B5 (Thermo Scientific) supplemented with dialyzed FCS (Thermo Scientific). This medium did not affect normal growth of the cells. Cells were differentiated with 50 µM retinoic acid (RA, Sigma) for 48–72 hours. Differentiated SHSY-5Y cells were plated in poly-Lysine (for this poly-D or poly-L-lysine was used, resulting in an identical outcome) coated culture dishes and cell morphology and neurite formation were quantified after 48 hours respectively. Neurite outgrowth was quantified as a percentage of highly β-III-tubulin positive cells bearing at least one projection exceeding two lengths of the cell body.

### Immunoblotting

Equal numbers of *Drosophila* S2 cells were centrifuged, washed once with PBS and lysed immediately in 1× Laemmli Sample Buffer (62,5 mM Tris/HCl pH 6,8; 2% SDS; 10% glycerol; bromophenol blue) with 10 mM NaF. SHSY-5Y cells were washed 1 time with PBS in tissue culture dishes and removed from the surface by scraping the cells into PBS. After centrifugation cells were lysed in 1× Laemmli Sample Buffer with 10 mM NaF. All sample were further sonicated and boiled for 4 min with 5% β-mercaptoethanol. Protein extracts were run on 12,5% SDS-Page gels, transferred onto nitrocellulose membranes and blocked with 5% milk in PBS/0,1% Tween, following by an overnight incubation with primary antibodies. The following antibodies were used: rabbit anti-dPANK/Fbl [5], rabbit anti-Twinstar [20], rabbit anti-cofilin and rabbit anti-phospho-cofilin (Cell Signaling), rabbit anti-phosphorylated AMP-activated kinase (Cell Signaling #2535) and mouse anti-tubulin (Sigma). HRP-conjugated secondary antibodies (Amersham) were used and detection was performed using enhanced chemiluminescence. Band intensities were quantified with Adobe Photoshop.

### Immunofluorescence

For immunofluorescence *Drosophila* S2 cells were seeded on concanavalin A-coated glass microscope slides and allowed to spread for 45 min. SHSY-5Y cells were plated on poly- lysine-coated glass slides (for this poly-D or poly-L-lysine was used, resulting in an identical outcome) and cultured for 48 hours. Cells were fixed with 3,7% formaldehyde for 15 minutes, washed 3 times with PBS and permeabilized with 0,2% Triton X-100 for 10 minutes. Slides were further blocked with 3% BSA in PBS/0,1% Tween for 30 minutes, followed by the 2 hour-incubation with primary antibody (mouse anti-beta-III-tubulin (clone 2G10, Sigma)). Secondary goat anti-mouse-Alexa488 antibodies (Molecular Probes) were used according to the manufacturer instructions (1 hour incubation). To visualize filamentous actin, cells were stained for 20 minutes with 20 U/mL rhodamine phalloidin (Molecular Probes) diluted with 3% BSA in PBS/0,1% Tween. Finally, microscope slides were mounted using CitiFluor mounting medium (Citifluor Ltd).

### Fluorescent microscopy, live cell imaging and image quantification

Fluorescent images were obtained with a Leica fluorescent microscope and processed with Leica software and Adobe Photoshop. More than 150 cells (from at least 10 different microscopic fields) per condition were counted to quantify the cell spreading (S2 cells) and neurite formation (SHSY-5Y cells).

Phase contrast live cell imaging was performed on a Zeiss 780 confocal microscope with a 63× glycerol immersion lens. S2 cells were seeded in ConA coated glass bottom culture dishes MatTek Corporation), imaged for 20 minutes per movie in 20 second intervals. Images were processed and kymographs created with ImageJ. For this, squares of 5–10 pixels thickness were drawn across the cell edges and the individual images combined to generate the kymographs. Representative cells, movies and kymographs are shown for each experiment.

### Statistical analysis

All results were obtained in at least two independent experiments, with a duplo or triplo of each experimental condition. Statistical significance was calculated using the Student's *t*-test (two-tailed, with equal variance). *p* values<0,05 were considered significant and were indicated as follows: p<0,05 - *; p<0,01 - **; p<0,001 - ***. Graph error bars represent S.E.M.

## Supporting Information

Figure S1 **Inhibition of dPANK activity in S2 cells induces an increase in phospho-Twinstar levels after 4 days of RNAi treatment.** (**A**) S2 cells were treated with control dsRNA or dPANK/fbl dsRNA (as described in Materials and Methods) to downregulate dPANK/Fbl protein levels and the cells were lysed 4 or 7 days after RNAi treatment. Immunoblots of whole cell extracts were incubated with an antibody against dPANK/Fbl, Tsr and p-Tsr. Tubulin was used as a loading control. (B) The graph illustrates the quantified levels of p-Tsr relative to untreated control cells.
(TIF)

Figure S2 **Serum starvation or oligomycin does not lead to increased levels of phospho-Twinstar.** S2 cells were left untreated, serum starved for 5 hours or treated with 100 nM oligomycin for 15 or 60 minutes. Immunoblots of whole cell extracts were probed for p-Tsr levels and the starvation effect (ATP depletion) was confirmed with an antibody against phosphorylated AMP-activated kinase. Tubulin was used as loading control. CTRL = control/untreated cells.
(TIF)

Figure S3 **SHSY-5Y cells differentiate and show morphological changes upon treatment with retinoic acid.** (**A–C**) Control SHSY-5Y cells were differentiated with RA, plated on poly-lysine and analyzed after 24 hours for their morphology (using rhodamin-phalloidin marking F-actin, red) and differentiation status (using β-III-Tubulin staining, green). In non treated SHSY-5Y cells no β-III-Tubulin positive cells were observed (data not shown). In RA-treated cultures undifferentiated and differentiated cells were visible. Undifferentiated cells showed very short filopodia, no filamentous actin was observed and β-III-Tubulin was not expressed (**A** and marked with white arrow heads in **B**). After 24 hours RA treatment most differentiated cells showed lower β-III-Tubulin expression associated with strong filamentous actin fibers (purple arrow heads) and an angular cell shape (**B,C**). 48 hours after seeding many differentiated cells showed an elongated shape, neurite growth and high expression of β-III-Tubulin (**Figure 5C–D** **, main text**). The presence of β-III-Tubulin positive neurites was used to quantify the effect of HOPAN on differentiation by RA and to quantify the rescue potential of pantethine (Figure 5D–G, main text).
(TIF)

Movie S1 **CoA deficient** ***Drosophila*** **S2 cells fail to form actin-based lamella.** dPANK/Fbl depleted S2 cells were seeded in concanavalin A coated glass bottom culture dishes and imaged during cell spreading and lamella formation in phase contrast with an inverted Zeiss confocal microscope using a 63× glycerol-immersion lens. Frames were acquired every 20 seconds for 20 minutes.
(AVI)

Movie S2 **Pantethine rescues actin-based lamella formation of dPANK/fbl depleted** ***Drosophila*** **S2 cells.** Pantethine treated dPANK/Fbl depleted S2 cells were seeded in concanavalin A coated glass bottom culture dishes and imaged during cell spreading and lamella formation in phase contrast as described under Figure S4.
(AVI)

Movie S3 **Control** ***Drosophila*** **S2 cells form actin-based lamella.** S2 cells treated with control RNAi constructs were seeded in concanavalin A coated glass bottom culture dishes and imaged during cell spreading and lamella formation in phase contrast as described under Figure S4.
(AVI)

Movie S4 **Pantethine does not affect actin-based lamella formation in control S2 cells.** S2 cells treated with control RNAi constructs and pantethine were seeded in concanavalin A coated glass bottom culture dishes and imaged during cell spreading and lamella formation in phase contrast as described under Figure S4.
(AVI)

Table S1 **Primers used for RNAi constructs.** The following primers were used to generate dsRNAs. 5′ T7 RNA polymerase binding site (TAATACGACTCACTATAGGG) was proceeding each primer's sequence.
(TIF)
