# Supplementary figures and images for: Cofilin/Twinstar Phosphorylation Levels Increase in Response to Impaired Coenzyme A Metabolism
Source: PLoS One. 2012 Aug 17;7(8):e43145. doi: 10.1371/journal.pone.0043145 (PMC3422318; doi:10.1371/journal.pone.0043145)

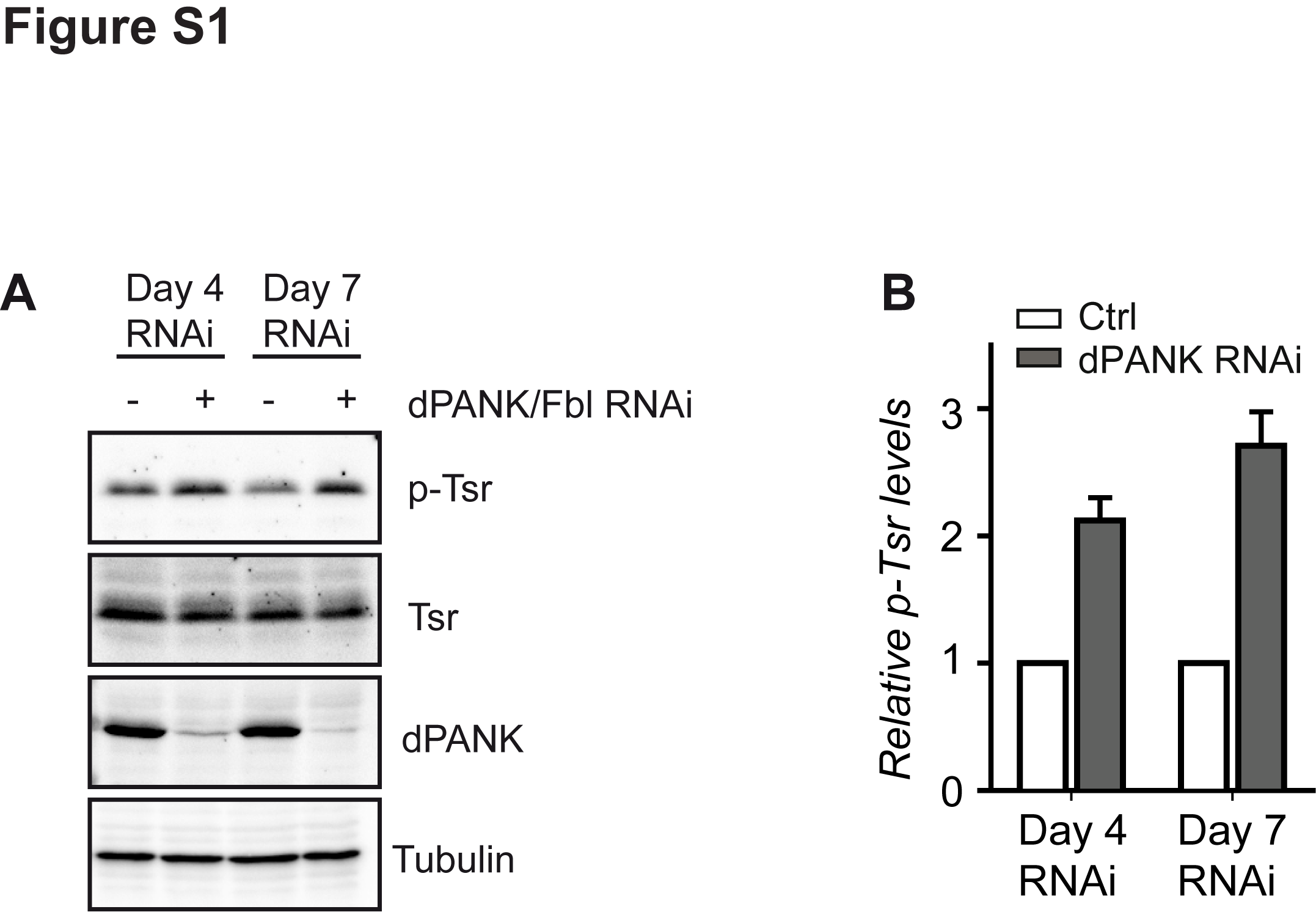

Supplement: Figure S1 — Inhibition of dPANK activity in S2 cells induces an increase in phospho-Twinstar levels after 4 days of RNAi treatment. (A) S2 cells were treated with control dsRNA or dPANK/fbl dsRNA (as described in Materials and Methods) to downregulate dPANK/Fbl protein levels and the cells were lysed 4 or 7 days after RNAi treatment. Immunoblots of whole cell extracts were incubated with an antibody against dPANK/Fbl, Tsr and p-Tsr. Tubulin was used as a loading control. (B) The graph illustrates the quantified levels of p-Tsr relative to untreated control cells. (TIF) [file pone.0043145.s001.tif]

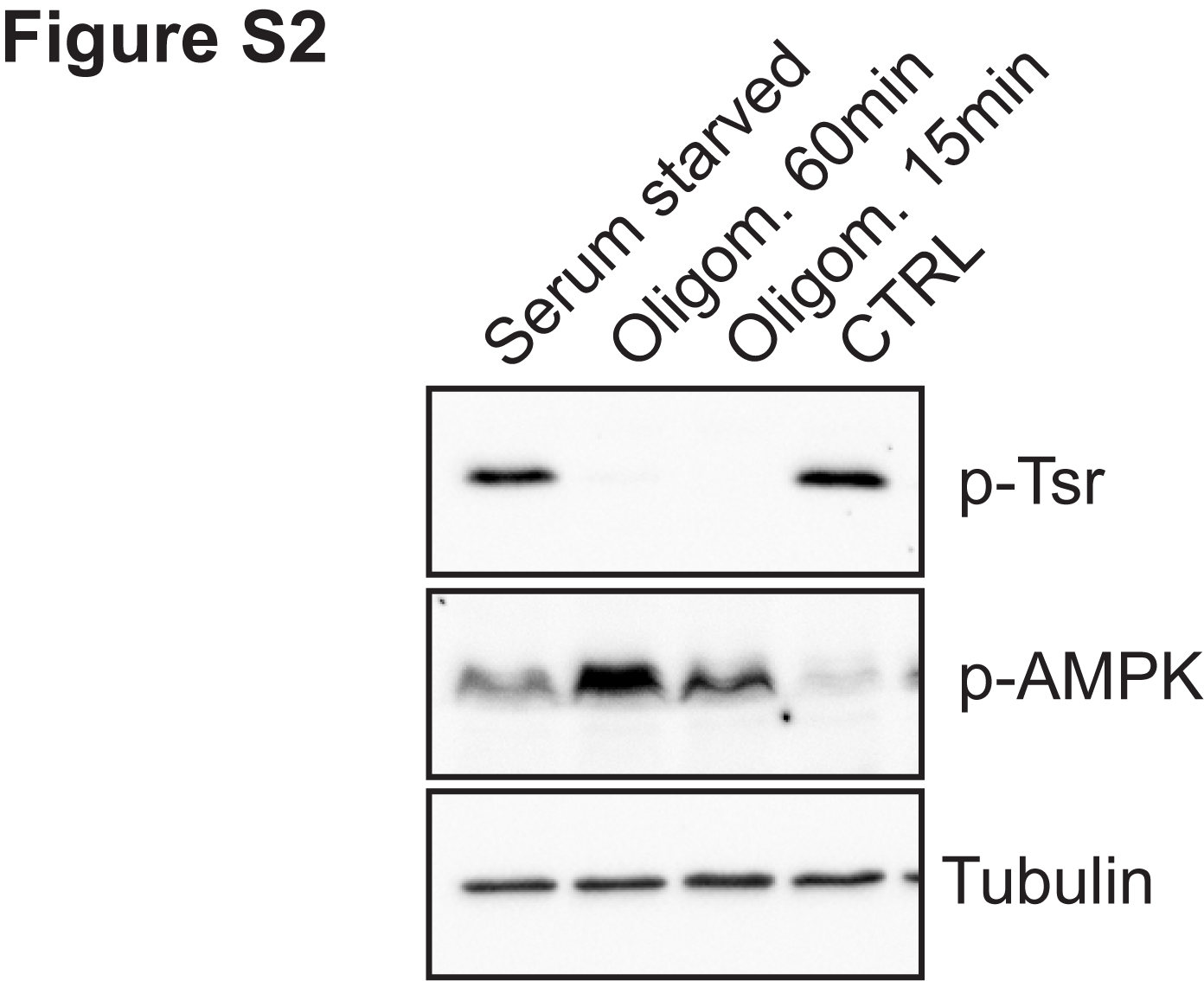

Supplement: Figure S2 — Serum starvation or oligomycin does not lead to increased levels of phospho-Twinstar. S2 cells were left untreated, serum starved for 5 hours or treated with 100 nM oligomycin for 15 or 60 minutes. Immunoblots of whole cell extracts were probed for p-Tsr levels and the starvation effect (ATP depletion) was confirmed with an antibody against phosphorylated AMP-activated kinase. Tubulin was used as loading control. CTRL = control/untreated cells. (TIF) [file pone.0043145.s002.tif]

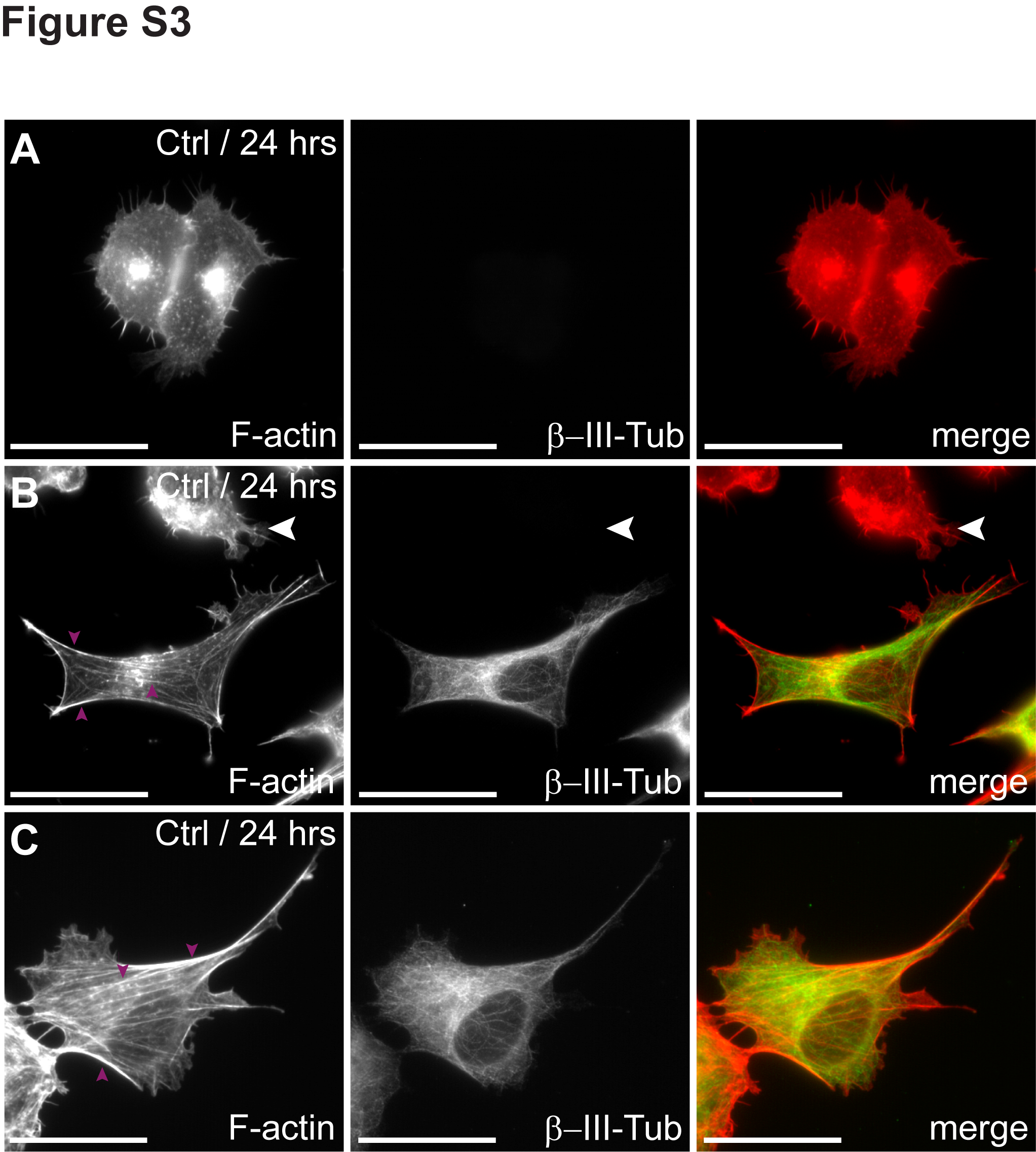

Supplement: Figure S3 — SHSY-5Y cells differentiate and show morphological changes upon treatment with retinoic acid. (A–C) Control SHSY-5Y cells were differentiated with RA, plated on poly-lysine and analyzed after 24 hours for their morphology (using rhodamin-phalloidin marking F-actin, red) and differentiation status (using β-III-Tubulin staining, green). In non treated SHSY-5Y cells no β-III-Tubulin positive cells were observed (data not shown). In RA-treated cultures undifferentiated and differentiated cells were visible. Undifferentiated cells showed very short filopodia, no filamentous actin was observed and β-III-Tubulin was not expressed (A and marked with white arrow heads in B). After 24 hours RA treatment most differentiated cells showed lower β-III-Tubulin expression associated with strong filamentous actin fibers (purple arrow heads) and an angular cell shape (B,C). 48 hours after seeding many differentiated cells showed an elongated shape, neurite growth and high expression of β-III-Tubulin ( Figure 5C–D , main text). The presence of β-III-Tubulin positive neurites was used to quantify the effect of HOPAN on differentiation by RA and to quantify the rescue potential of pantethine (Figure 5D–G, main text). (TIF) [file pone.0043145.s003.tif]

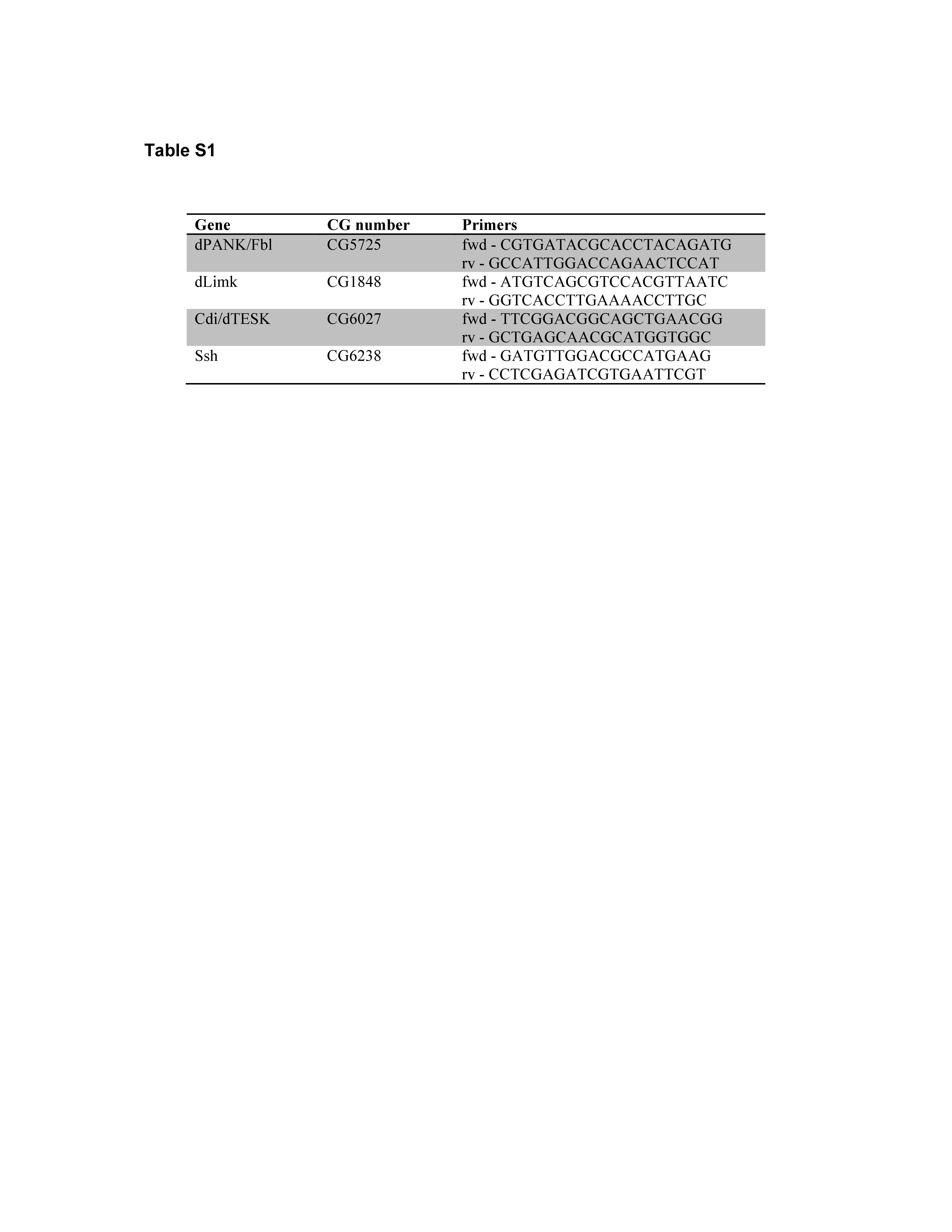

Supplement: Table S1 — Primers used for RNAi constructs. The following primers were used to generate dsRNAs. 5′ T7 RNA polymerase binding site (TAATACGACTCACTATAGGG) was proceeding each primer's sequence. (TIF) [file pone.0043145.s008.tif]
